# A Rapid and Specific Assay for the Detection of MERS-CoV

**DOI:** 10.3389/fmicb.2018.01101

**Published:** 2018-05-29

**Authors:** Pei Huang, Hualei Wang, Zengguo Cao, Hongli Jin, Hang Chi, Jincun Zhao, Beibei Yu, Feihu Yan, Xingxing Hu, Fangfang Wu, Cuicui Jiao, Pengfei Hou, Shengnan Xu, Yongkun Zhao, Na Feng, Jianzhong Wang, Weiyang Sun, Tiecheng Wang, Yuwei Gao, Songtao Yang, Xianzhu Xia

**Affiliations:** ^1^Animal Science and Technology College, Jilin Agricultural University, Changchun, China; ^2^Key Laboratory of Jilin Province for Zoonosis Prevention and Control, Institute of Military Veterinary, Academy of Military Medical Sciences, Changchun, China; ^3^College of Veterinary Medicine, Jilin University, Changchun, China; ^4^Jiangsu Co-innovation Center for Prevention and Control of Important Animal Infectious Disease and Zoonoses, Yangzhou, China; ^5^State Key Laboratory of Respiratory Disease, Guangzhou Institute of Respiratory Heath, The First Affiliated Hospital of Guangzhou Medical University, Guangzhou, China; ^6^Guangzhou Eighth People’s Hospital of Guangzhou Medical University, Guangzhou, China; ^7^Department of Clinical Laboratory, College of Medicine, Sir Run Run Shaw Hospital, Zhejiang University, Hangzhou, China

**Keywords:** Middle East respiratory syndrome coronavirus, reverse transcription loop-mediated isothermal amplification, nucleic acid visualization, visual detection, RT-LAMP-VF

## Abstract

Middle East respiratory syndrome coronavirus (MERS-CoV) is a novel human coronavirus that can cause human respiratory disease. The development of a detection method for this virus that can lead to rapid and accurate diagnosis would be significant. In this study, we established a nucleic acid visualization technique that combines the reverse transcription loop-mediated isothermal amplification technique and a vertical flow visualization strip (RT-LAMP-VF) to detect the N gene of MERS-CoV. The RT-LAMP-VF assay was performed in a constant temperature water bath for 30 min, and the result was visible by the naked eye within 5 min. The RT-LAMP-VF assay was capable of detecting 2 × 10^1^ copies/μl of synthesized RNA transcript and 1 × 10^1^ copies/μl of MERS-CoV RNA. The method exhibits no cross-reactivities with multiple CoVs including SARS-related (SARSr)-CoV, HKU4, HKU1, OC43 and 229E, and thus exhibits high specificity. Compared to the real-time RT-PCR (rRT-PCR) method recommended by the World Health Organization (WHO), the RT-LAMP-VF assay is easy to handle, does not require expensive equipment and can rapidly complete detection within 35 min.

## Introduction

Coronaviruses (CoVs, family *Coronaviridae*, subfamily *Coronavirinae*) can infect a wide variety of animals, including humans, avians, rodents, carnivores, chiropters and other mammals, and can cause respiratory, enteric, hepatic, and neurological diseases (Ge et al., 2016; Xu et al., 2016). CoVs exhibit a high frequency of recombination and high mutation rates, which may allow them to adapt to new hosts and ecological niches (Woo et al., 2009). Currently, six human coronaviruses (HCoVs) have been identified, including, HCoV-229E, HCoV-OC43, HCoV-NL63, HCoV-HKU1, severe acute respiratory syndrome (SARS)-CoV and Middle East respiratory syndrome (MERS)-CoV. Most HCoVs can cross species barriers, including SARS-CoV and MERS-CoV (Peiris et al., 2003; Widagdo et al., 2017).

Middle East respiratory syndrome coronavirus has been classified as a lineage C betacoronavirus, and its structure comprises a ∼30.1 kb single-stranded positive-sense RNA genome that is closely related to the lineage C betacoronaviruses of *Tylonycteris* bat CoV HKU4 and *Pipistrellus* bat CoV HKU5 (Zaki et al., 2012). Some evidence demonstrates that dromedary camels are a major reservoir host for MERS-CoV, and virus from infected dromedary camels can be transmitted across species to infect humans (Azhar et al., 2014; Ling et al., 2015). Additionally, the virus can be transmitted human-to-human by close contact (Nazer, 2017). As of the 26th of January 2018, 2143 laboratory-confirmed cases of MERS-CoV infection have been reported, and these cases include 750 deaths globally. Because no available commercial vaccines or specific treatments currently exist, rapid and accurate diagnosis is significant for the prevention of extensive MERS outbreaks.

Current diagnostic tests for MERS-CoV include reverse transcriptase polymerase chain reaction (RT-PCR), real-time reverse transcription PCR (rRT-PCR), reverse transcription loop-mediated isothermal amplification (RT-LAMP), and real-time RT-LAMP (Corman et al., 2012; Lu et al., 2014; Shirato et al., 2014; Bhadra et al., 2015; Chan et al., 2015). However, the areas in which the application of these methods are limited because the results are analyzed with electronic equipment, such as agarose gel electrophoresis set up, real-time quantitative instrumentation and turbidimeter. Thus, a nucleic acid visualization method that requires no special equipment that combines RT-LAMP with a vertical flow visualization strip (RT-LAMP-VF) was established to detect MERS-CoV nucleic acids.

## Materials and Methods

### The Rational of RT-LAMP-VF

**FIGURE 1:**
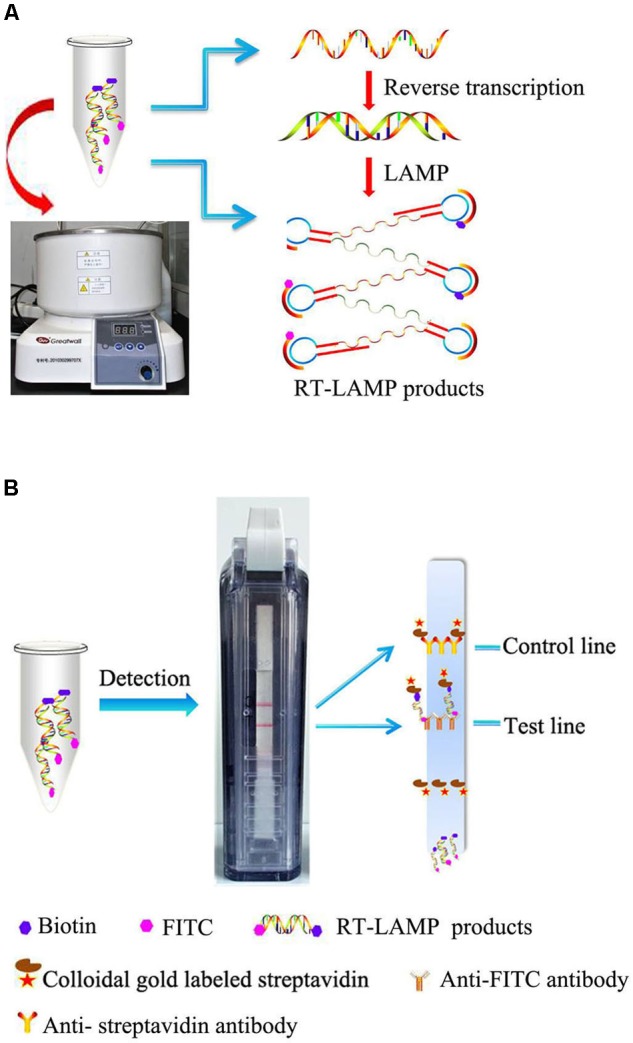
Schematic illustration of the RT-LAMP-VF assay. **(A)** RT-LAMP was performed in a constant temperature water bath. **(B)** RT-LAMP products were detected with a vertical flow visualization strip.

In the RT-LAMP-VF assay, the RNA of MERS-CoV was amplified by RT-LAMP using multiple cross-linked primers (six primers) which target the conserved region of N gene. Two loop primers (LF and LB) which are involved in isothermal amplification are labeled with fluorescein isothiocyanate (FITC) and biotin, respectively. After amplification, the amplicons are simultaneously labeled with FITC and biotin. The amplicons that are labeled with biotin can bind to colloidal gold particles conjugated with streptavidin to form a complex. Then the complex, labeled with FITC, is captured by an anti-FITC antibody that is coated on the text line of the strip, and the test results are presented as a colored line that is visible to the naked eye (**Figure 1**).

### Virus and RNA Extraction

Total RNA from Huh7 cells infected with the China GD01 strain (GenBank Accession No. KT006149) of MERS-CoV was kindly provided by professor Jincun Zhao from the Guangzhou Institute of Respiratory Health (Wang et al., 2015). The China GD01 strain was cultured in Huh7 cell monolayers at a 0.1 multiplicity of infection (MOI). The cell culture lysates were collected 30 h post-infection and used for MERS-CoV RNA extraction with the QIAamp viral RNA minikit. All the work with infectious MERS-CoV was conducted in the Guangzhou Institute of Respiratory Disease of Biosafety Level 3 (BSL3) Laboratory.

The intestinal tissues of bats infected with SARS-related (SARSr)-CoV or HKU4 were gifted by professor Changchun Tu from the Institute of Military Veterinary Medicine. RNA was extracted using a commercial RNA extraction kit (RNeasy Mini Kit, Qiagen, Hilden, Germany) in the BSL2 Laboratory.

### Primer Design

**FIGURE 2:**
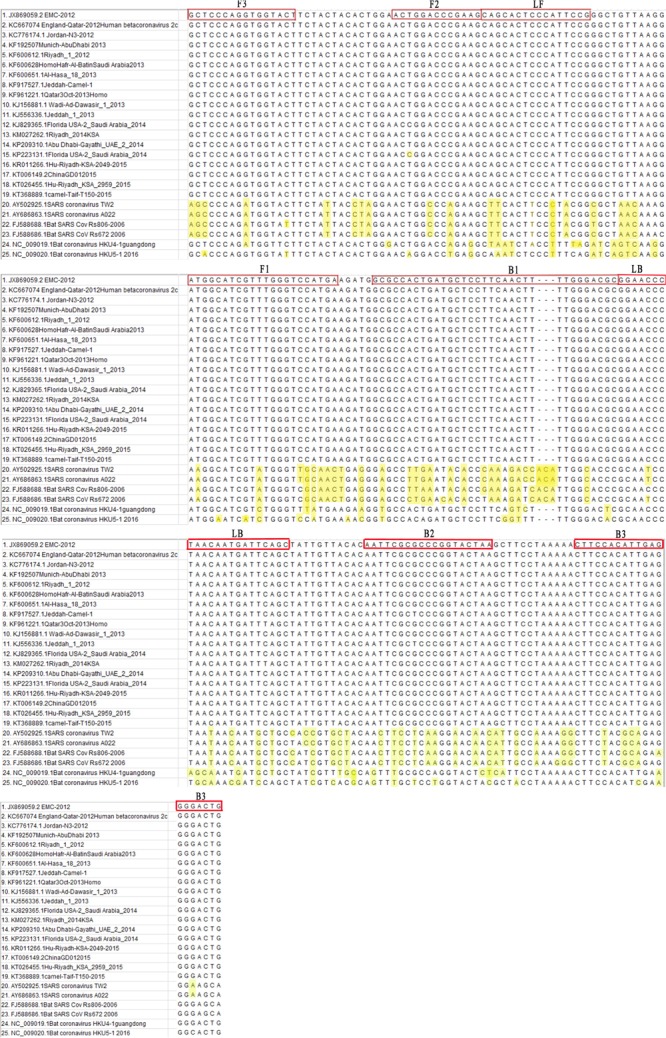
Primer positions of the RT-LAMP-VF assay. The MERS-CoV N gene was retrieved from GenBank and aligned using MEGA7 software.

In total, the N gene sequences from 35 representative complete MERS-CoV strains from previous studies that were collected from different regions between 2012 and 2015 were aligned. Due to the picture size limit, alignment results that included 19 MERS-CoV strains, 2 SARS-CoV strains, 1 HKU4 strain, 1 HKU5 strain, and 2 SARSr-CoV strains were presented in **Figure 2**. A 213-nt region among the complete RNA positions 28848–29061 of the EMC strain (GenBank Accession No. JX869059) was selected for submission to an online primer design software (Primer Explorer V4).^1^ Six specific primers were designed in eight regions of the gene (**Table 1**) and synthesized by Shanghai Invitrogen Biological Engineering, Co., Ltd.

**Table 1:** The primer set for the MERS-CoV RT-LAMP assay.

Primers name	Primers position	Sequence (5′–3′)
F3	28848–28866	GCTCCCAGGTGGTACTTCT
B3	29061–29042	CAGTCCCCTCAATGTGGAAG
FIP (F1c+F2)	28939–28918+ 28872–28890	TCATGGACCCAAACGATGCCATACTGG AACTGGACCCGAAG
BIP (B1c+B2)	28956–28977+ 29029–29011	GCTCCTTCAACTTTTGGGACGCTTAGTA CCGGGCGCGAATT
LF	28906–28891	FITC-CGGAATGGGAGTGCTG
LB	28978–29000	Biotin-GGAACCCTAACAATGATTCAGC

*A 213-nt region between EMC strain RNA positions 28848–29061 was selected for the design of the RT-LAMP-VF primers. The 5′ ends of the LF and LB primers were labeled with FITC and biotin, respectively.*

### Pretreatment of the Recombinant Plasmid and Synthesized RNA Transcripts of the MERS-CoV N Gene

A pcDNA3.1 (+) recombinant plasmid (build by predecessors of our team) containing the MERS-CoV N gene (GenBank Accession No. JX869059) was measured with a spectrophotometer, and the copy number of the recombinant plasmid was calculated using the following formula: copies/μl = 6.02 × 10^23^× 10^-9^× concentration/(fragment length × 660). We obtained the recombinant plasmid concentration of 370 ng/μl, which corresponded to 7.9 × 10^10^ copies/μl, and 10-fold serial dilutions of the recombinant plasmid ranging from 10^7^ to 10^0^ copies/μl were prepared.

The MERS-CoV N gene target sequence (GenBank Accession No. JX869059) was synthesized using *in vitro* transcription by Bao Biological, Co., Ltd., Dalian, China. The RNA transcripts were then purified and quantified, and the copy number was calculated using the following formula: copies/μl = 6.02 × 10^23^× 10^-9^× concentration/(fragment length × 340). The concentration of the synthesized RNA transcript was 1760 ng/μl as measured with a spectrophotometer, which corresponded to 9.7 × 10^12^ copies/μl. The synthesized RNA transcripts were stored at -80°C after purification.

### RT-LAMP-VF Assay Reaction and Product Detection

The reactions were prepared as previously described (Notomi et al., 2000). Briefly, 25 μl reaction mixtures comprising 5 μl of template, 8 μM of MgSO_4_, 0.2 μM of FIP and BIP, 0.1 μM of LF and LB, 0.05 μM of F3 and B3, 1.4 μM of deoxynucleotide triphosphates (dNTPs) (Thermo Scientific), 0.2 M of betaine and 8 U of Bst DNA polymerase (New England BioLabs) were used. If the recombinant plasmid was used as the template, the mixture (all the components except the enzyme) was heated to 95°C for 5 min and then immediately placed on ice. Next, 8 U of Bst DNA polymerase was added, and the mixture was incubated at 65°C for 50 min. If the synthesized RNA transcripts were used as the template, 5 U of avian myeloblastosis virus reverse transcriptase (AMV) (Bioer Technology, Co., Ltd., Hangzhou, China) was additionally added to the reaction mixture described above, and the mixture was incubated at 65°C for 50 min.

The reactions were performed at five different temperatures (61, 63, 65, 67, or 69°C) for 50 min, and the results were analyzed with the vertical flow visualization strip (Ustar Biotech, Co., Ltd., Hangzhou, China) to obtain the optimal reaction temperature. Three replications were performed for each trial.

Based on the optimum reaction temperature, the reaction time (30, 40, or 50 min) and inner primer concentration (0.2 or 0.4 μM) were simultaneously optimized. Three replications were performed for each trial.

Because this method was ultimately applied to detect MERS-CoV RNA, the recombinant plasmid was replaced with synthesized RNA transcripts as the amplification template. The reaction process was executed in a thermostatic water bath at varying temperatures (59, 61, 63, or 65°C) for 30 min. Three replications were performed for each trial.

### RT-LAMP-VF Assay Specificity and Sensitivity Evaluation

Multiple respiratory pathogen nucleic acids were extracted from the NATtrol^TM^sp RP Multimarker Controls kit (ZeptoMetrix Corporation, Franklin, MA, United States). The pathogens that made up RP Multimarker 1 (RP1) and RP Multimarker 2 (RP2) controls included HKU-1, OC43, NL63, 229E, influenza A/B, rhinovirus, adenovirus, and parainfluenza, etc. (**Table 2**). RNA of the RP1 controls, RP2 controls, SARSr-CoV and HKU4 and synthesized RNA transcripts were used to evaluate the specificity of the RT-LAMP-VF assay. Three replications were performed for each trial.

**Table 2:** Respiratory pathogens included in the NATtrol^TM^ sp RP Multimarker controls.

(a)		(b)	
RP1 Respiratory virus	Strain	RP2 Respiratory virus	Strain
Influenza A H3N2	Brisbane/10/07	Influenza A H1	New Caledonia/20/99
Influenza A H1N1	NY/02/2009	Influenza B	Florida/02/06
Rhinovirus	Type 1A	RSV	Type A
Adenovirus	Type 3	Parainfluenza	Type 2
Parainfluenza	Type 1	Parainfluenza	Type 3
Parainfluenza	Type 4	Coronavirus	HKU-1 (recombinant)
Metapneumovirus	Peru 6-2003	Coronavirus	OC43
*C. pneumoniae*	CWL-029	Coronavirus	NL63
*M. pneumoniae*	M129	Coronavirus	229E
*Coxsackievirus*	Type A1	*Bordetella pertussis*	A639

*(a) RP1 kit; (b) RP2 kit*.

Ten-fold serial dilutions of the synthesized RNA transcripts (ranging from 2 × 10^6^ to 2 × 10^0^ copies/μl) were subjected to the RT-LAMP-VF assay to assess the detection limits. Three replications were performed for each trial.

### Using MERS-CoV Nucleic Acids to Evaluate the RT-LAMP-VF Assay

Middle East respiratory syndrome coronavirus RNA in the total RNA from the infected cells was quantified by absolute quantification rRT-PCR assay using the One-step PrimeScript™ RT-PCR Kit (TaKaRa Biotechnology, Co., Ltd., China). The primes and probe were designed and synthesized by TaKaRa Biotechnology, Co., Ltd., China (Supplementary Table 1). Each 25 μl reaction was formulated according to the manufacturer’s instructions. Amplification was performed in an Applied Biosystems Agilent StrataGene MX3005P real-time PCR instrument (Agilent Technologies, Co., Ltd., United States). A negative control was included in all the runs to monitor assay performance, and different copies of synthesized RNA transcripts were used as standard samples. Three replications were performed for each trial.

Next, 10-fold serial dilutions of MERS-CoV RNA were determined by the RT-LAMP-VF method to assess the detection limits. Three replications were performed for each trial.

The total RNA of throat swabs collected from healthy people was purified using a commercial RNA extraction kit according to the manufacturer’s instructions. After mixing the total RNA of the throat swabs with different copy numbers of the MERS-CoV RNA at a 9:1 volume ratio, the mixtures were subjected to the RT-LAMP-VF assay. Simultaneously, the total RNA from the throat swabs harvested from healthy people served as the control. Three replications were performed for each trial.

### Sensitivity Comparison of Conventional RT-LAMP, rRT-PCR, and RT-LAMP-VF

The diluted MERS-CoV RNAs were used as the template. The conventional RT-LAMP reaction mixture and the amplification were mixed and performed under the same conditions as the RT-LAMP-VF assay. The products of the conventional RT-LAMP were analyzed using 2% agarose gel electrophoresis. Three replications were performed for each trial.

The rRT-PCR assay against upE and N of MERS-CoV were performed according to the report of Lu et al. (2014), and the One Step PrimeScript™ RT-PCR Kit was used. Detailed information about the primers and probe were provided in Supplementary Table 2. The thermal cycling involved 30 min at 42°C, followed by 90 s at 95°C and then 45 cycles of 95°C for 15 s, 60°C for 40 s. Each run included one no-template control. A positive test result was defined as a well-defined exponential fluorescence curve that crossed the threshold within 45 cycles. Three replications were performed for each trial.

### Ethics Statement

The throat swabs were collected at the Institute of Military Veterinary Medicine in accordance with the approved guidelines and relevant regulations. Written informed consent was obtained from all the subjects prior to their participation in the study. The Ethics Committee and Institutional Review Board of Use Committee of the Chinese People’s Liberation Army (No. SYXK2009-045) approved all the experimental procedures.

## Results

### Optimizing the RT-LAMP-VF Reaction Conditions

First, recombinant plasmids were used as the template to optimize the RT-LAMP-VF assay reaction conditions. To screen for the optimum temperature, a denaturation step was initially performed at 95°C for 5 min, and the reaction was then incubated at five different temperatures (61, 63, 65, 67, or 69°C) for 50 min. **Table 3** revealed that the highest amplification efficiency occurred at 65°C. Analysis of the results revealed no significant differences in three replications. Therefore, 65°C was deemed the optimum temperature for the RT-LAMP-VF assay.

**Table 3:** Reaction temperature optimization for RT-LAMP.

Temperature/°C	Recombinant plasmids dilution (2 × copies/μl)								
	10^7^	10^6^	10^5^	10^4^	10^3^	10^2^	10^1^	10^0^	*N*
61	+	+	+	+	+	+	-	-	-
63	+	+	+	+	+	+	-	-	-
65	+	+	+	+	+	+	+	-	-
67	+	+	+	+	+	+	-	-	-
69	+	+	+	+	+	+	-	-	-

*The serially diluted 10-fold recombinant plasmids were detected at five different temperatures for 50 min. Three replications were performed for each trial*.

To determine the optimal amplification time for the RT-LAMP-VF assay, recombinant plasmids were detected at three different reaction times (30, 40, or 50 min), and the application signal was monitored by the strip. As presented in **Table 4**, the same results were obtained at incubation periods of 30 and 40 min in equivalent conditions. Thus, 30 min was selected as the optimal reaction time. Simultaneously, the primer concentrations were optimized as presented in **Table 4**, and 0.2 μM for FIP/BIP, 0.1 μM for LF/LB and 0.05 μM for F3/B3 were deemed optimal.

**Table 4:** Reaction times and concentrations for inner primer optimization for RT-LAMP.

Time/min	FIP/BIP concentration (μM)	Recombinant plasmid dilution (2 × copies/μl)								
		10^7^	10^6^	10^5^	10^4^	10^3^	10^2^	10^1^	10^0^	*N*
30	0.2	+	+	+	+	+	-	-	-	-
	0.4	+	+	+	+	+	+	-	-	-
40	0.2	+	+	+	+	+	+	-	-	-
	0.4	+	+	+	+	+	+	-	-	-
50	0.2	+	+	+	+	+	+	+	-	-
	0.4	+	+	+	+	+	+	+	-	-

*The serially diluted 10-fold recombinant plasmids were detected at three different times at 65°C. Moreover, the amplification was performed at different concentration for each of inner primers FIP and BIP (0.2 or 0.4 μM). Three replications were performed for each trial*.

The feasibility of the RT-LAMP-VF assay was further confirmed using synthesized RNA transcripts in the optimized reaction conditions. As presented in **Table 5**, the best sensitivity and specificity were obtained when the reaction was performed at 61°C.

**Table 5:** Reaction temperature optimization for RT-LAMP.

Temperature/°C	Synthesized RNA transcript dilution (2 × copies/μl)								
	10^7^	10^6^	10^5^	10^4^	10^3^	10^2^	10^1^	10^0^	*N*
65	+	+	+	+	-	-	-	-	-
63	+	+	+	+	+	-	-	-	-
61	+	+	+	+	+	+	-	-	-
59	+	+	+	+	+	+^a^	-	-	-

*^a^The result was weakly positive. The serially diluted 10-fold synthesized RNA transcripts were detected at four different temperatures for 30 min. Three replications were performed for each trial*.

### Specificity and Sensitivity of the RT-LAMP-VF Assay

**FIGURE 3:**
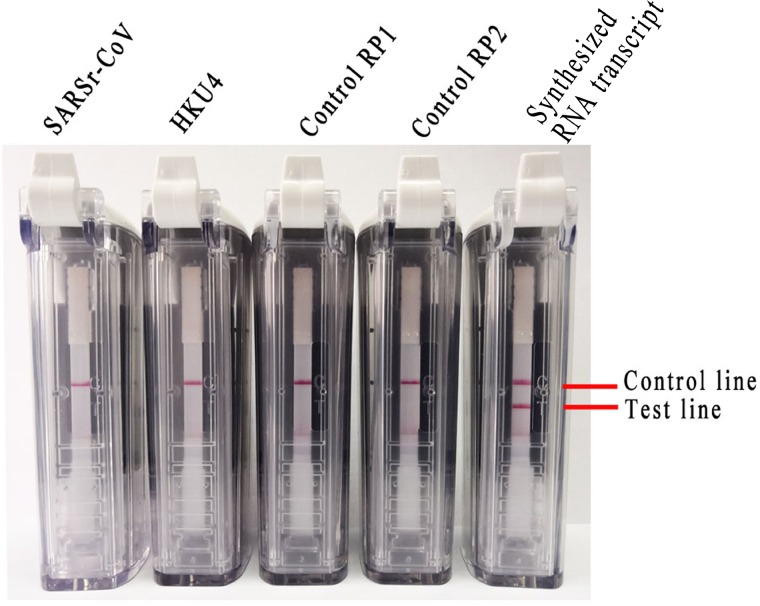
Specificity of the RT-LAMP-VF assay as analyzed by extracting RNA from multiple respiratory pathogens and synthesized RNA transcripts.

Living organisms infected by respiratory pathogens often exhibit atypical respiratory symptoms. To accurately screen MERS-CoV positive patients with respiratory symptoms, multiple respiratory pathogen nucleic acids produced from RP1 and RP2, SARSr-CoV RNA, HKU4 RNA and synthesized RNA transcripts were used to evaluate the specificity of the RT-LAMP-VF assay. As illustrated in **Figure 3**, only the synthesized RNA transcripts tested positive, and multiple respiratory pathogen nucleic acids from RP1 and RP2, SARSr-CoV, and HKU4 all tested negative with the RT-LAMP-VF assay. Therefore, the RT-LAMP-VF assay had no cross-reactivity with multiple CoVs, including SARSr-CoV, HKU4, HKU1, OC43 and 229E, etc.

**FIGURE 4:**
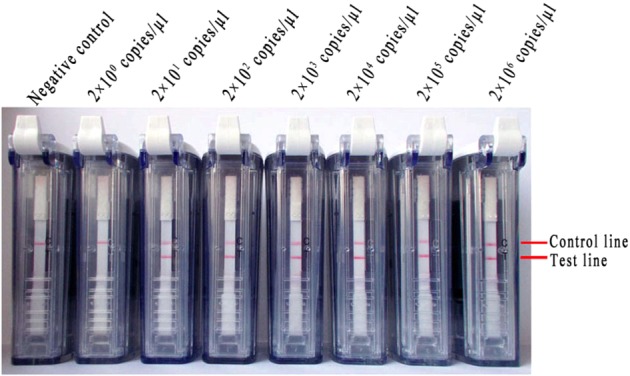
Detection of the RT-LAMP-VF assay limit using a series of synthesized RNA transcripts.

Next, 10-fold dilutions of synthesized RNA transcripts (ranging from 2 × 10^6^ to 2 × 10^0^ copies/μl) were used to assess the sensitivity of the RT-LAMP-VF assay. The assay limit of detection for synthesized RNA transcripts was 2 × 10^1^ copies/μl within 35 min. The representative results of the RT-LAMP-VF assay for serial dilutions of synthesized RNA transcripts were presented in **Figure 4**.

### Using MERS-CoV Nucleic Acids to Evaluate the RT-LAMP-VF Assay

Absolute quantification rRT-PCR was used to measure the quantity of MERS-CoV RNA in total RNA. The linear amplification of standard samples was achieved across a 7-log dynamic range, from 5 × 10^1^ to 5 × 10^8^ copies per reaction, with a calculated efficiency value of 99.1% (Supplementary Figure 1). Next, the MERS-CoV RNA copy number was calculated as 1.5 × 10^7^ copies/μl in 3.4 × 10^8^ copies/μl of total RNA using the standard curve linear formula regarding the relationship between the copy number and cycles.

**FIGURE 5:**
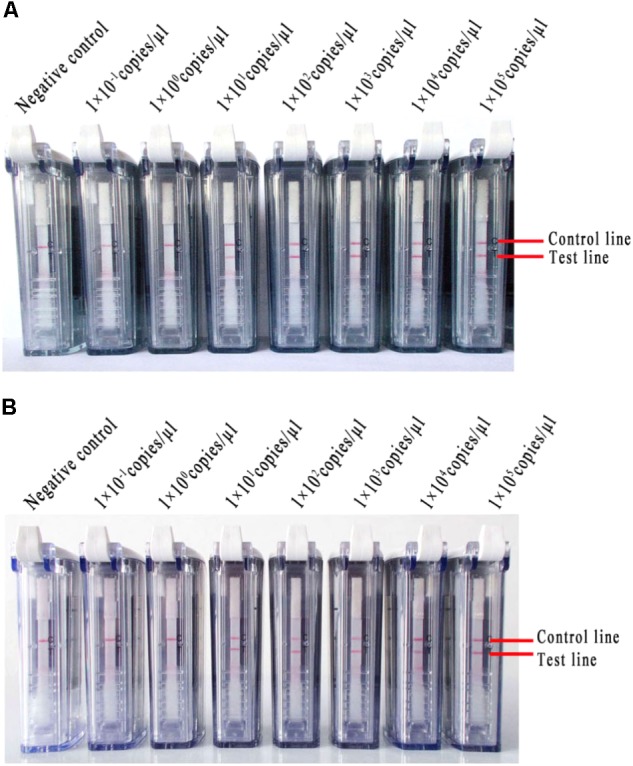
The RNA of MERS-CoV was used to evaluate the RT-LAMP-VF assay. **(A)** Ten-fold serial dilutions of MERS-CoV RNA were detected with the RT-LAMP-VF assay. **(B)** The RT-LAMP-VF assay was performed on mixtures in which the dilutions of MERS-CoV RNA were spiked into the total RNA of throat swabs collected from healthy people.

Ten-fold serial dilutions of MERS-CoV RNA (ranging from 1 × 10^5^ to 1 × 10^-1^ copies/μl) were detected with the RT-LAMP-VF assay. Negative results were observed when the concentration was lower than 1 × 10^1^ copies/μl (**Figure 5A**).

Different copy numbers of viral RNA (ranging from 1 × 10^5^ to 1 × 10^-1^ copies/μl) were spiked into the total RNA of the throat swabs collected from healthy people, and the mixtures were used to further evaluate the RT-LAMP-VF assay. This assay did not react non-specifically with nucleic acids from the throat swabs. Additionally, the limit of detection was equivalent to 1 × 10^1^ copies/μl of MERS-CoV RNA (**Figure 5B**), which indicated that the total throat swab RNA did not affect the specificity or sensitivity of the RT-LAMP-VF assay for detecting MERS-CoV.

### Sensitivity Comparison of Conventional RT-LAMP, rRT-PCR, and RT-LAMP-VF

**FIGURE 6:**
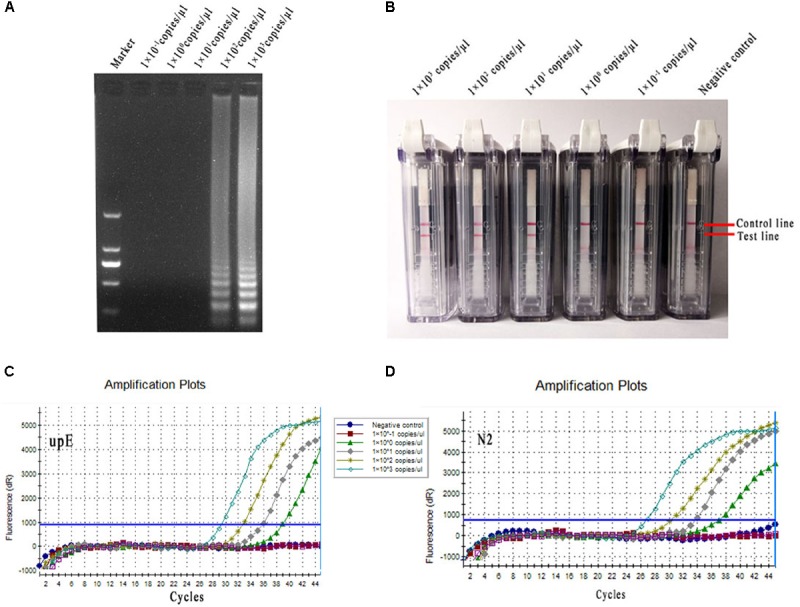
Sensitivity comparison of conventional RT-LAMP, rRT-PCR, and RT-LAMP-VF. **(A)** Conventional RT-LAMP assay. **(B)** The RT-LAMP-VF assay. **(C)** The rRT-PCR assay for upE. **(D)** The rRT-PCR assay for N2.

The diluted MERS-CoV RNAs (1 × 10^3^–1 × 10^-1^ copies/μl) were used as the templates in conventional RT-LAMP, rRT-PCR and RT-LAMP-VF assays. The positive reaction was clearly observed for the 1 × 10^2^ copies/μl of MERS-CoV RNA sample using 2% agarose gel electrophoresis in the conventional RT-LAMP reaction (**Figure 6A**). The limit of detection was equivalent to 1 × 10^1^ copies/μl of MERS-CoV RNA in the RT-LAMP-VF (**Figure 6B**). Moreover, both of the rRT-PCR assays could detect templates as little as 1 × 10^0^ copies/μl of MERS-CoV RNA for the upE or N2 gene (**Figures 6C,D**), and these results are consistent with those of previous reports.

## Discussion

Middle East respiratory syndrome coronavirus is widely distributed throughout 27 countries and regions, and the numbers of annual infections and deaths continue to grow, especially in the Middle East. Because no licensed effective vaccines or treatment methods exist, timely diagnosis and isolation are the most effective methods to control viral outbreak. LAMP technology is a promising method for nucleic acid detection. Under constant temperature conditions, nucleic acids can be rapidly amplified using specific primers with advantages that include high specificities and short detection times. Currently, LAMP technology has been widely used for the detection of West Nile virus, influenza virus, yellow fever virus, Marburg virus, Ebola virus, Zika virus, and other virulent viruses (Kurosaki et al., 2010; Ge et al., 2013; Kwallah et al., 2013; Cao et al., 2016; Xu et al., 2016; Chotiwan et al., 2017). In the field of MERS-CoV detection, Bhadra et al. (2015) established a real time quantitative method, i.e., a one-step strand displacement RT-LAMP, which can detect 5–50 PFU/ml of MERS-CoV. Shirato et al. (2014) established a real-time quantitative RT-LAMP assay for the N gene by adding pyrophosphate or nucleic acid dyes to the amplification system. Lee et al. (2016) developed a real time one-pot RT-LAMP method to target the N gene. These methods are relatively feasible, specific and rapid molecular diagnostic technologies.

Despite the advantages of the RT-LAMP application, there are still some technical shortcomings, such as false positive problems and the signal-reading equipment that is required. Thus, these methods are not widely applied in grassroot laboratories. In this article, an RT-LAMP-VF assay for the detection of the MERS-CoV N gene was established. In contrast with previous RT-LAMP methods, six primers are used in the reaction mixture which enormously increases the amplification efficiency. Additionally, two loop primers (LF and LB) are labeled with FITC or biotin, creating the visible results on the test strip.

The optimal amplification temperatures were different for the recombinant plasmids and synthesized RNA transcripts, which could have been due to several reasons. First, the RT-LAMP-VF assay requires AMV Reverse Transcriptase to reverse transcribe the RNA into cDNA. AMV Reverse Transcriptase exhibits its highest activity at 42–55°C, while Bst DNA polymerase operates best at 65°C. The higher reaction temperature likely decreased the efficiency of reverse transcription. Therefore, when synthesized RNA transcripts were used as the template, the temperature was lowered to facilitate reverse transcription. Furthermore, the supercoiled structures of the recombinant plasmids were steady, thus making it necessary to maintain the helix state at a high temperature.

According to a study by Notomi et al. (2000), inserting TTTT into the FIP and BIP primers can enhance LAMP efficiency. However, the inner primers utilized in many studies of the detection of nucleic acids with LAMP assays do not contain TTTT sequences (Notomi et al., 2000; Mori et al., 2001; Mekata et al., 2006; Wang et al., 2013; Ge et al., 2017). To investigate the efficiency of the RT-LAMP-VF assay after inserting TTTT into the FIP and BIP primers, two sets of inner primers were designed (Supplementary Table 3); one set contained the TTTT sequence and one set did not. Under the same amplification conditions, the primer set containing the TTTT sequence produced better results (Supplementary Figure 2), which is consistent with the conclusion drawn by Lee et al. (2016).

The rRT-PCR assay is recommended by the WHO for the routine confirmation of cases of MERS-CoV infection. In this study, the two rRT-PCR assays were guided by the study reports of Lu et al. (2014), and the results were compared with those of conventional RT-LAMP and RT-LAMP-VF assays in terms of sensitivity. The limit of detection was equivalent to 1 × 10^1^ copies/μl of MERS-CoV RNA for the RT-LAMP-VF, which is more sensitive than the conventional RT-LAMP reaction (1 × 10^2^ copies/μl). Here, we observed high sensitivities, the two rRT-PCR assays detected as litter as 1 × 10^0^ copies/μl in both the upE and N2 assays. Although the sensitivity is lower than that of the rRT-PCR, the RT-LAMP-VF assay only requires 35 min, which is far faster than the rRT-PCR assay, which requires 2 h. Moreover this method does not require electronic equipment to read the signal of the amplification products.

Because the collected clinical MERS samples were mostly sputum, we needed to verify that the RT-LAMP-VF assay did not react with sputum nucleic acids and that the sputum samples did not affect the assay sensitivity. However, clinical specimens infected with MERS-CoV are difficult to obtain in non-endemic countries such as China. The mixtures of the total RNA of throat swabs and different copy numbers of MERS-CoV RNA were subjected to the RT-LAMP-VF assay. Our results indicated that the sensitivity and specificity of this method were not interfered with by other components in the clinical sample.

In summary, our study demonstrates that the RT-LAMP-VF assay enriches the practical tools that are available molecular diagnosis of MERS-CoV. The RT-LAMP-VF assay for MERS-CoV exhibited no cross-reactivity with multiple CoVs, which demonstrated good specificity. Additionally, this assay’s rapidity and lack of need for special instrumentation indicates that it can be easily adapted to different laboratory settings.

## Author Contributions

HW, SY, and XX designed the experiments. PH, HJ, ZC, HC, FY, XH, FW, CJ, PFH, SX, YZ, JW, WS, and TW performed the experiments. HW, JZ, BY, NF, YG, SY, and XX analyzed the data. PH, HW, and HJ wrote the manuscript.

## Conflict of Interest Statement

The authors declare that the research was conducted in the absence of any commercial or financial relationships that could be construed as a potential conflict of interest. The reviewer TS and handling Editor declared their shared affiliation.

## Footnotes

1 http://primerexplorer.jp/e/
