# Supplementary material for: A Rapid and Specific Assay for the Detection of MERS-CoV
Source: Front Microbiol. 2018 May 29;9:1101. doi: 10.3389/fmicb.2018.01101 (PMC5987675; doi:10.3389/fmicb.2018.01101)
Supplement: Supplementary file 1 [file Data_Sheet_1.docx]

Supplementary Material

A rapid and specific assay for the detection of MERS-CoV

Pei Huang*, Hualei Wang, Zengguo Cao, Hongli Jin, Hang Chi, Jincun Zhao, Beibei Yu, Feihu Yan, Xingxing Hu, Fangfang Wu, Cuicui Jiao, Pengfei Hou, Shengnan Xu, Yongkun Zhao, Na Feng, Jianzhong Wang, Weiyang Sun, Tiecheng Wang, Yuwei Gao, Songtao Yang, Xianzhu Xia

*** Correspondence:** Hualei Wang: [whl831125@163.com](mailto:whl831125@163.com)

# Supplementary Tables and Figures

## Supplementary Figures

**Supplementary Table 1: Primers and probe sequences for the absolute quantitation rRT-PCR assay**

| Primer or probe name | Genome location | Sequence (5′ – 3′) |
| --- | --- | --- |
| MERS-F | 28853-28870 | CAGGTGGTACTTCTACTA |
| MERS-R | 28983-29000 | AGCTGAATCATTGTTAGG |
| MERS-P | 28873-28892 | /6-FAM/CTGGAACTGGACCCGAAGCA/BHQ1/ |

**Supplementary Table 2: Primers and probes sequences for the MERS-CoV rRT-PCR assay**

| Genome target | Genome location | Primer or probe | Primer or probe |
| --- | --- | --- | --- |
| upE | 27458–27475 | Forward primer | GCAACGCGCGATTCAGTT |
|  | 27549–27530 | Reverse primer | GCCTCTACACGGGACCCATA |
|  | 27477–27502 | Probe | /6-FAM/CTCTTCACATAATCGCCCCGAGCTCG/BHQ1/ |
| N2 | 29424–29442 | Forward primer | GGCACTGAGGACCCACGTT |
|  | 29498–29477 | Reverse primer | TTGCGACATACCCATAAAAGCA |
|  | 29445–29471 | Probe | /6-FAM/CCCCAAATTGCTGAGCTTGCTCCTACA/BHQ1/ |

The primer and probe sequences are from a report by Lu et al. ([Lu et al., 2014](#_ENREF_14)).

**Supplementary Table 3: Inner primer sequences**

| Groups | Primers name | Sequence (5′ – 3′) |
| --- | --- | --- |
| T1 | FIP(F1c+F2) | TCATGGACCCAAACGATGCCAT(TTTT)ACTGGAACTGGACCCGA  AG |
|  | BIP(B1c+B2) | GCTCCTTCAACTTTTGGGACGC(TTTT)TTAGTACCGGGCGCGAA  TT |
| T2 | FIP(F1c+F2) | TCATGGACCCAAACGATGCCATACTGGAACTGGACCCGAAG |
|  | BIP(B1c+B2) | GCTCCTTCAACTTTTGGGACGCTTAGTACCGGGCGCGAATT |

## Supplementary Figures


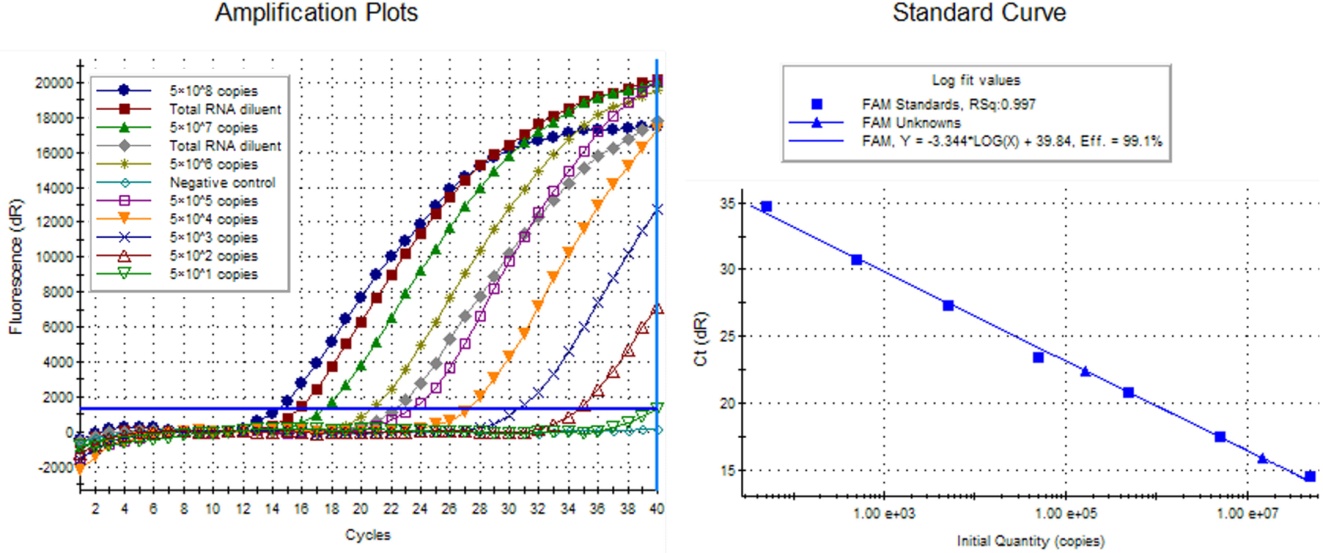


**Supplementary Figure 1**: MERS-CoV RNA quantitatively analyzed with the absolute quantification rRT-PCR assay.
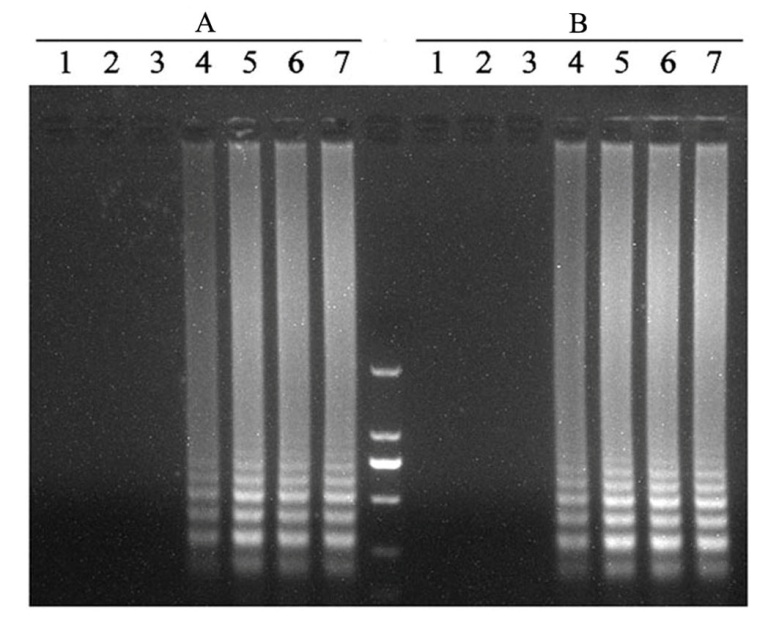


**Supplementary Figure 2: Influence of the TTTT structure in the inner primer on LAMP amplification.** A: Inner primers with the TTTT structure. B: Inner primers without the TTTT structure. 1: Negative control; 2: 2×10^1^ copies/µl; 3: 2×10^2^ copies/µl; 4: 2×10^3^ copies/µl; 5: 2×10^4^ copies/µl; 6: 2×10^5^ copies/µl; 7: 2×10^6^ copies/µl.
